# Supplementary material for: Splice-Junction-Based Mapping of Alternative Isoforms in the Human Proteome
Source: Cell Rep. Author manuscript; Available in PMC 2020 Jan 15. (PMC6961840; doi:10.1016/j.celrep.2019.11.026)
Supplement: 2 [file NIHMS1546469-supplement-2.zip › DF1/PXD006675/PulmonaryArtery/PulmonaryArtery_5_SVIL_DSEGDTPSLINWPSSK.pdf]

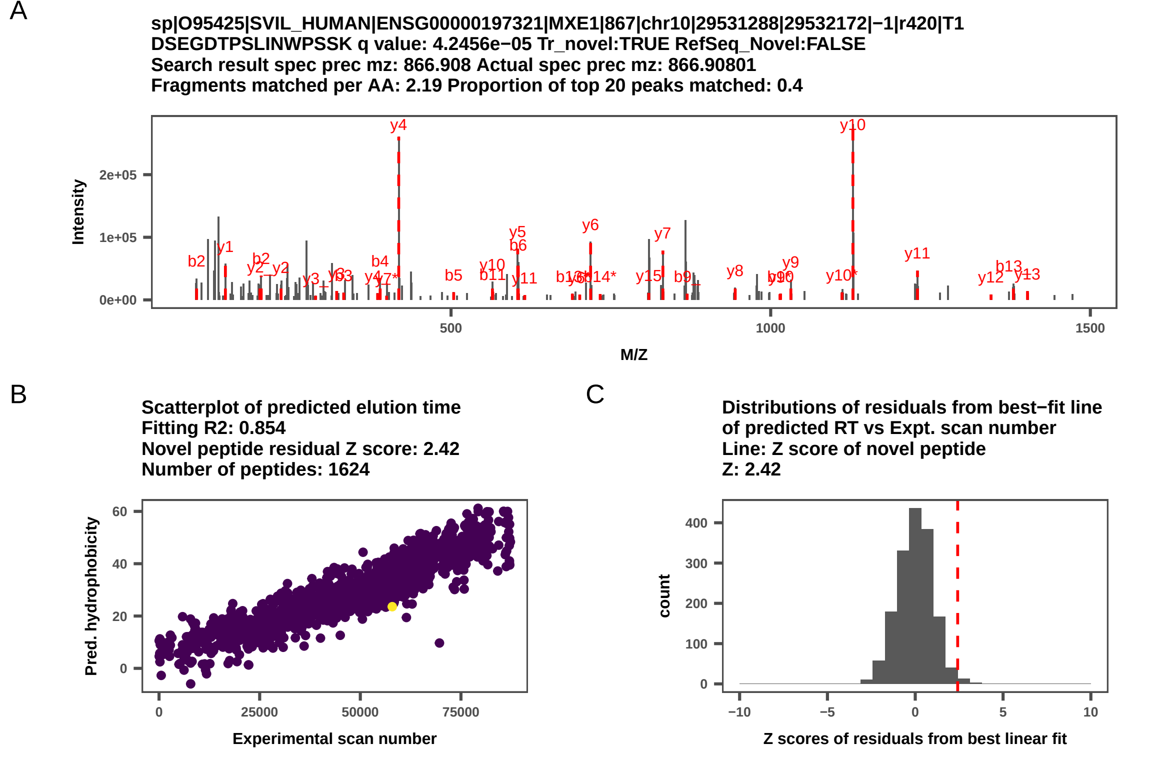
A
sp|O95425|SVIL_HUMAN|ENSG00000197321|MXE1|867|chr10|29531288|29532172|−1|r420|T1
DSEGDTPSLINWPSSK q value: 4.2456e−05 Tr_novel:TRUE RefSeq_Novel:FALSE
Search result spec prec mz: 866.908 Actual spec prec mz: 866.90801
Fragments matched per AA: 2.19 Proportion of top 20 peaks matched: 0.4
Intensity
2e+05
1e+05
0e+00
500
1000
1500
M/Z
B
Scatterplot of predicted elution time
Fitting R2: 0.854
Novel peptide residual Z score: 2.42
Number of peptides: 1624
Pred. hydrophobicity
60
40
20
0
0
25000
50000
75000
Experimental scan number
C
Distributions of residuals from best−fit line
of predicted RT vs Expt. scan number
Line: Z score of novel peptide
Z: 2.42
count
400
300
200
100
0
−10
−5
0
5
10
Z scores of residuals from best linear fit
